# Giant Magnetoresistance Biosensors for Food Safety Applications

**DOI:** 10.3390/s22155663

**Published:** 2022-07-28

**Authors:** Shuang Liang, Phanatchakorn Sutham, Kai Wu, Kumar Mallikarjunan, Jian-Ping Wang

**Affiliations:** 1 Department of Chemical Engineering and Materials Science, University of Minnesota, Minneapolis, MN 55455, USA; liang822@umn.edu; 2 Department of Food Science and Nutrition, University of Minnesota, St. Paul, MN 55108, USA; sutha074@umn.edu; 3 Department of Electrical and Computer Engineering, University of Minnesota, Minneapolis, MN 55455, USA; 4 Department of Electrical and Computer Engineering, Texas Tech University, Lubbock, TX 79409, USA

**Keywords:** foodborne pathogen, toxin, food safety, biosensor, giant magnetoresistance

## Abstract

Nowadays, the increasing number of foodborne disease outbreaks around the globe has aroused the wide attention of the food industry and regulators. During food production, processing, storage, and transportation, microorganisms may grow and secrete toxins as well as other harmful substances. These kinds of food contamination from microbiological and chemical sources can seriously endanger human health. The traditional detection methods such as cell culture and colony counting cannot meet the requirements of rapid detection due to some intrinsic shortcomings, such as being time-consuming, laborious, and requiring expensive instrumentation or a central laboratory. In the past decade, efforts have been made to develop rapid, sensitive, and easy-to-use detection platforms for on-site food safety regulation. Herein, we review one type of promising biosensing platform that may revolutionize the current food surveillance approaches, the giant magnetoresistance (GMR) biosensors. Benefiting from the advances of nanotechnology, hundreds to thousands of GMR biosensors can be integrated into a fingernail-sized area, allowing the higher throughput screening of food samples at a lower cost. In addition, combined with on-chip microfluidic channels and filtration function, this type of GMR biosensing system can be fully automatic, and less operator training is required. Furthermore, the compact-sized GMR biosensor platforms could be further extended to related food contamination and the field screening of other pathogen targets.

## 1. Introduction

Foodborne infections and food waste are major global challenges with significant threats to human health and the environment. Each year, nearly 48 million people in the United States become ill, 128,000 people are hospitalized and 3,000 people die due to foodborne infections (Data source: The Centers for Diseases Control and Prevention, https://www.cdc.gov/foodborneburden/2011-foodborne-estimates.html, accessed on 1 July 2022). Generally, foodborne diseases are caused by the consumption of food that is contaminated with foodborne pathogens and/or their toxins [1,2]. The pathogens that cause the most foodborne illnesses include *Norovirus*, *Salmonella* spp., *Listeria monocytogenes*, *Clostridium perfringens*, *Campylobacter* spp., *Staphylococcus aureus*, *Escherichia coli O157:H7*, and Shiga toxin-producing *Escherichia coli* (STEC) [3]. Not only the foodborne illnesses due to pathogenic spoilage microorganisms are a severe threat to human health, but the loss of food at different stages of the food supply chain caused by spoilage can also be economically destructive. The Food and Agricultural Organization (FAO) estimates around one-third of all produced foods (1.3 billion tons of edible food) for human consumption is lost and wasted each year throughout the supply chain, which costs approximately USD 750 billion per year (Data source: FAO, https://www.fao.org/news/story/en/item/196220/icode/, accessed on 1 July 2022). Due to the presence of foodborne illness, food spoilage, and food waste, it is essential to develop rapid and sensitive detection methods for early detection of foodborne pathogens to quickly diagnose the presence of pathogens from the food products and along food production lines, ensure the quality and safety of food, and minimize the occurrence of foodborne diseases.

Nowadays, various techniques have been developed to detect and identify specific pathogens and toxins present in foods. Conventional cell culture is generally a standard method for foodborne pathogen detection in the food industry due to its high sensitivity, reliability, and cost-effectiveness [4]. However, the entire process which includes pre-enrichment, isolation, and identification is lengthy and labor-intensive [5]. Advanced technologies, including immunological assays such as enzyme-linked immunosorbent assays (ELISA) and molecular biology-based methods such as polymerase chain reaction (PCR), provide a comprehensive genetic profile and identify the specific microorganism [6,7]. However, the major limitation of these methods is that they may cause false-positive results [8,9]. Moreover, the preparation steps are tedious, expensive, and require highly skilled technicians [10]. Recently, the rapidly evolving field of biosensors has provided alternative approaches to pathogen and toxin detection in foods [11]. The biosensors that are commonly used for the detection of foodborne pathogens include but are not limited to optical, electrochemical, mass-based biosensors, and magnetoresistive biosensors [1].

**Figure 1:**
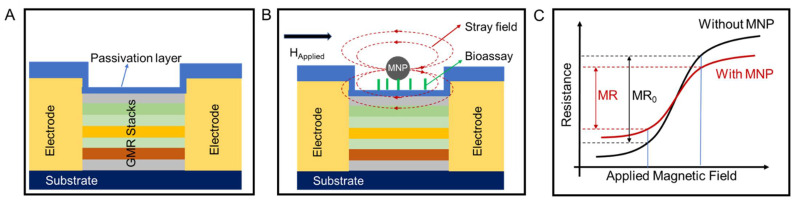
Schematics of (**A**) a typical GMR biosensor, (**B**) a functionalized GMR biosensor under an applied field, and (**C**) R-H curves of a GMR biosensor with and without conjugated MNPs on the surface.

Among various biosensors for detecting foodborne pathogens/toxins, biosensors based on the giant magnetoresistance (GMR) stand out for their high sensitivity, cost-efficiency, and the ability for multiplexed assays [12,13,14,15]. The GMR effect was first observed in Fe/Cr multilayer structures by Fert et al. and Grünberg et al. in 1988 and soon became a popular research topic due to its great potential in the field of hard drives, memories, sensors, etc. [16,17,18,19]. In 1998, Baselt et al. firstly reported the GMR-based biosensors [20]. To date, GMR biosensors have been used in various biomedical areas, such as the detection of cancer biomarkers and viruses, as well as monitoring brain and cardiac activities [21,22,23,24]. As shown in Figure 1A, a typical GMR biosensor generally has a stack structure consisting of GMR thin films, passivation layers, and electrodes. The multi-layered GMR thin film structure exhibits magnetoresistance (MR), which is the change in electrical resistance in response to the change of externally applied magnetic field. The passivation layers on top of GMR stacks can prevent the corrosion caused by interfacing with biofluids. GMR biosensors detect the magnetic nanoparticle (MNP)-label analytes for quantitative assays. These MNPs are usually functionalized with protein or nucleic acid probes that can specifically bind to target analytes and be captured to the GMR sensor surface via different immunoassay methods (e.g., sandwich assay, competitive assay, etc.). Under an applied magnetic field, the MNPs that are in the vicinity of the GMR sensor surface will generate stray fields, which alters the MR of the sensor, as shown in Figure 1B,C. Theoretical models for the effect of stray fields generated by magnetic beads have been established and compared to experimental results [25,26,27,28]. Generally, the more the MNPs conjugated to the sensor surface, the larger change in resistance (i.e., the difference between MR and MR_0_) can be observed. Quantitative detection of the MNPs, and thus the biomarkers, can then be done by recording the change in the sensor resistance.

In the past five years, several review articles on biosensors for food safety applications have been published, but these reviews are mostly related to electrochemical, fluorometric, or mass-sensitive sensors [29,30,31]. As a novel biosensing platform, there are a few reviews on GMR biosensors, yet the GMR sensors for food safety are rarely discussed [32,33,34]. Therefore, the objective of this review is to give a detailed discussion about GMR biosensing platforms in the area of food safety applications. In Section 2, current technologies for food safety applications are introduced as a comparison to the GMR biosensing platforms. The details about GMR sensor structures, bioassay strategies, and GMR sensing platforms are discussed in Section 3. In terms of food safety applications, GMR-based biosensing platforms have been demonstrated to be capable of detecting foodborne pathogens, toxins, heavy metal contaminates, livestock viruses, as well as food allergens. Examples of these applications are given in Section 4. Section 5 summarizes and concludes the content of this review. The current challenges and future opportunities of GMR biosensors in food safety applications are commented on in Section 6.

## 2. Current Technologies for Food Safety Applications

### 2.1. The Conventional Culture Methods

**Figure 2:**
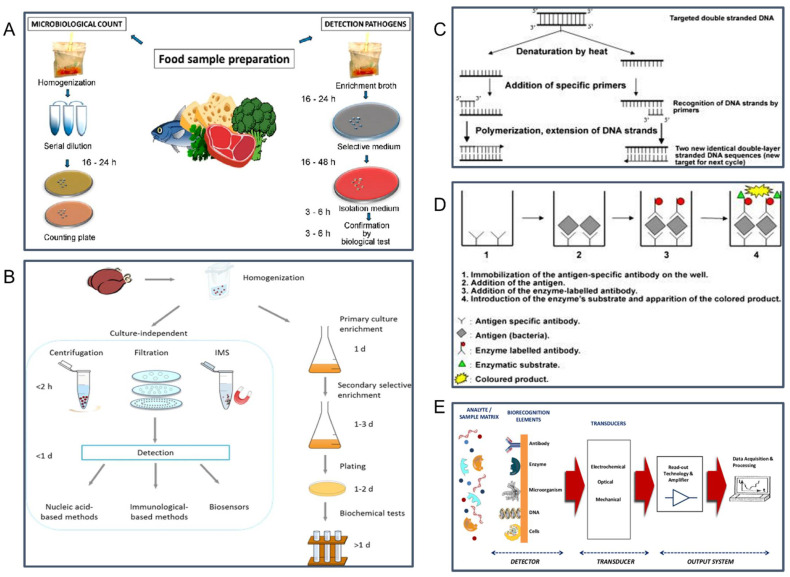
(**A**) Conventional cell culture and colony counting method for foodborne pathogen detection. (**B**) Cultureindependent vs. conventional culture enrichment methods for food pathogen detection. (**C**) Principle of PCR-based methods consists of different cycles of denaturation by heat, extension phasing using specific primers, and exponential amplification. (**D**) Principle of a typical “sandwich ELISA” technique. (**E**) Biosensor-based platforms. (**A**) is reprinted with permission from Ref. [62], under the terms of the Creative Commons Attribution License (CC BY). (**B**) is reprinted with permission from Ref. [63], 2015, Wiley. (**C**,**D**) is reprinted with permission from Ref. [39], 2018, Elsevier B.V. (**E**) is reprinted with permission from Ref. [58], under the terms of the Creative Commons Attribution License (CC BY).

The traditional method of cell culture and colony counting is considered to be the basic approach for pathogenic bacteria detection [4]. The culturing of microorganisms directly or after enrichment on agar media or selective media and counting the number of the colonies is highly efficient to detect live microorganisms [35]. A schematic illustration of the cell culture technique including microbiological count and pathogens detection techniques is presented in Figure 2A. This classic method has the advantages of low cost, high reliability, and stability, but this method can be excessively time-consuming as they depend on the ability of the microorganisms to grow in different media such as pre-enrichment media, selective enrichment media, and selective plating media [1]. Figure 2B compares culture enrichment steps and other culture-independent techniques for food pathogen detection. The entire processes of pre-enrichment, isolation, and identification can take several days to deliver results since it requires the microorganism to multiply to visible colonies [4]. In addition, the preparation of culture media, inoculation of plates, and colony counting make this method laborious [4]. The culture enrichment steps also contain detection challenges because of the complex food metrics, potential lack of homogeneity, and the possibility of a low number of pathogens within the matrix [36]. Since the location of pathogens varies for different food products, homogenization (e.g., ultrasound treatment) is required to achieve a complete product analysis [36]. In addition, various food matrix compounds, such as proteins, lipids, vitamins, and minerals, might need to be removed to prevent matrix interference [37]. Table 1 provides a summary of the major advantages and disadvantages of the cell culture methods and other methods for foodborne pathogen detection discussed in Section 2.

### 2.2. Polymerase Chain Reaction (PCR)

The PCR technique is one of the most commonly used nucleic acid-based assays for foodborne pathogen detection [10]. Due to its speed, accuracy, and relative ease, the PCR-based technique is also one of the United States Department of Agriculture (USDA)’s and the United States Food and Drug Administration (USFDA)’s preferred laboratory procedures for microbial analysis in foods. By identifying specific target DNA fragments that correspond to an organism’s genome sequence, PCR can detect as little as one bacterial pathogen present in food [38]. PCR-based methods contain different cycles of denaturation by heat, extension phase using specific primers and a thermostable polymerization enzyme, and exponential amplification [39,40]. Figure 2C shows a schematic illustration of one PCR cycle in a thermocycler. Law et al. reported several nucleic acid-based methods including simple PCR, multiplex PCR (mPCR), real-time/quantitative PCR (qPCR), loop-mediated isothermal amplification (LAMP), and nucleic acid sequence-based amplification (NASBA), and microarray technology [10]. PCR tests have been employed to detect various foodborne pathogens including *Salmonella* spp., *Listeria monocytogenes*, *Staphylococcus aureus*, *Escherichia coli O157:H7*, and *Campylobacter* spp. [41,42,43]. There are also numerous commercially available PCR kits for the detection of *Escherichia coli*, such as the BAX™ system (Hygiena LLC, Camarillo, CA, USA), R.A.P.I.D.™ LT real-time PCR systems (Idaho Technology Inc., Salt Lake City, UT, USA), iQ-Check™ (Bio-Rad Laboratories Inc., Hercules, CA, USA), TaqMan^®^ detection kit (Thermo Fisher Scientific Inc., Waltham, MA, USA) [44]. Ellinggson et al. developed a rapid real-time quantitative PCR for the detection of *Salmonella* spp. in ready-to-eat beef products in 2004 [45]. This method was able to detect one colony of *Salmonella* in 1 mL of the food product within 12 h. A control was performed with visual immunoprecipitate and cultural methods and a correlation of 100% was obtained. In 2007, Malorny et al. developed a duplex 5′ nuclease (TaqMan) real-time PCR for the detection of *Salmonella Enteritidis* in chicken carcass rinses and eggs [46]. The detection limit was less than 3 CFU/50 mL of carcass rinse or 10 mL of eggs. The sensitivity and specificity comparing the traditional culture-based method were both 100%. The PCR-based methods are rapid, highly sensitive, and selective [10]. PCR is also less time-consuming than culturing and plating, it takes from 5 to 24 h to produce a result but this depends on the type of specific PCR variants used [47]. However, the major limitation of the PCR method is it can cause false-positive or false-negative results [8]. Moreover, this high-quality DNA preparation requires expensive reagents and instrumentations and highly skilled technicians (see Table 1) [8].

### 2.3. Immunological Methods

The immunoassays work by detecting specific binding of antibodies and antigens which provide a wide range of targets (Wang et al., 2019) [48]. Different immunoassays have been used to detect foodborne pathogens, namely the Enzyme immunoassay (EIA), enzyme-linked fluorescent assay (ELFA), enzyme-linked immunosorbent assay (ELISA), and Immuno-magnetic beads (IMBs) [49,50,51,52]. The most widely used immunoassay is the ELISA technique [53]. Figure 2D exhibits the principle of the sandwich-ELISA, which is the most common immunoassay used [39]. ELISA takes advantage of the high specificity of the antibody, and the sensitivity of the enzyme assay and can detect multiple pathogens in 15 min [39]. In 2007, Magliulo et al. developed a multiplex sandwich chemiluminescent enzyme immunoassay for the simultaneous detection of *E. coli O157:H7*, *Yersinia enterocolitica*, *S. Typhimurium*, and *Listeria monocytogenes*, the detection limit was approximately 10^4^–10^5^ CFU/mL for all pathogens [54]. In general, immunoassays allow real-time detection of pathogens within less time compared to cultural methods. However, these methods have some drawbacks including potential interferences from chemical contaminants in food samples and complicated assay steps that require trained technicians (see Table 1) [55].

### 2.4. New Types of Biosensors

Due to the need to overcome the limitations of the traditional methods, efforts have been made to develop a rapid sensitive and simple pathogen detection process to protect the quality and safety of food products. Recently, new biosensors have become a more promising choice for rapid pathogen detection because of their fast, high sensitivity, low cost, and ease of use [11]. A biosensor is a device that produces a measurable signal from physical, chemical, or biological events to detect target analyses [56,57]. Biosensors typically consist of two main components including a target recognition component such as receptors, nucleic acids, or antibodies and a signal transducer that converts target recognition into detectable signals, as shown in Figure 2E [58]. Unlike PCR-based assays and immunoassays which require sample pre-enrichment for concentrating the pathogens before detection, biosensors do not require sample pre-enrichment which makes them relatively easy to operate [59]. Table 1 summarizes the major advantages and disadvantages of emerging new biosensors compared with other foodborne pathogens detection methods. In addition, biosensors provide rapid, on-site tracking, and real-time results throughout the production process [60]. In 2020, Ali et al. reported a review of the application of biosensors for foodborne pathogen detection in food products and the use of this technology in the food industry such as hygiene monitor, dairy products, meat products, and fish products [61]. Biosensors can be classified into several different groups depending on their working principles. Examples of biosensors based on the types of physical transducer include electrochemical, optical, and mechanical biosensors [1].

#### 2.4.1. Optical Biosensors

Optical methods are used to detect the optical change caused by the interaction between the target analyte and the biological recognition element and convert the measurable signal which corresponds to the concentration of the analyte in the sample [64]. Optical biosensors have several advantages including high sensitivity, specificity, label-free, and rapid detection [65]. Alamer et al. reported the common optical techniques employed fluorescence, chemiluminescence, colorimetric, and surface plasmon resonance (SPR) [66]. SPR is a commonly used optical detection platform for label-free biosensing [67]. Campbell and Kim reported that real-time binding analysis of SPR can be attained by acquiring the change of optical reflectivity with time since the shift of the resonance frequency is corresponding to the change in the surface concentration of species adsorbed on the metal surface [68]. Although SPR is well developed and is the most commonly used method among optical biosensors, the instrument is expensive and complicated to operate [69,70]. In addition, the major challenge of the SPR biosensors for the detection of pathogens in complex food samples is that the interfering effects can arise from the sample matrix, especially the non-specific adsorption of non-target molecules from the sample to the sensing surface [37].

#### 2.4.2. Electrochemical Biosensors

Electrochemical biosensing assays rely on the conversion of DNA base-pair recognition into an electrical signal [71]. Due to the advantages of low cost, high accuracy, and miniaturization capacity, electrochemical biosensors are among the most widely used platforms for foodborne pathogen detection [61,72]. Based on the detection signals produced by the presence of heavy metal ions in the solution matrix, electrochemical biosensors can be classified into impedimetric, potentiometric, amperometric, voltammetric, electrochemiluminescent, and conductometric methods [73]. Many researchers had reported successful foodborne pathogen detection by electrochemical biosensors. For example, a direct-charge transfer conductometric biosensor was used for the detection of *Bacillus cereus* in various food samples including alfalfa sprouts, strawberries, lettuce, tomatoes, fried rice, and cooked corn, the detection limit was 35–88 CFU/mL with an analysis time of 6 min [74]. De Ávila et al. described the use of an amperometric magneto immunosensor for the detection of *Staphylococcus aureus* with a detection limit of 1 CFU/mL and the detection time was 2 h [75]. Yamada et al. developed a single-walled carbon nanotube-based (SWCNT) device for portable and rapid detection of *E. coli* K12 and *Staphylococcus aureus* [76]. Simultaneous detection was performed in the dual pathogen solution with 10^2^ CFU/mL concentrations with an analysis time of 1 min. Sobhan et al. (2019) reported on the incorporation of SWCNT-based biosensors to detect *Yersinia enterocolitica* in Kimchi products [77]. The SWCNT sensor reacted specifically with the target bacteria with a low detection of 10^4^ CFU/mL.

#### 2.4.3. Mechanical Biosensors

Mechanical biosensors operate based on the detection of small changes in mass and utilize interaction between the target analyte and the immobilized biological recognition element on the functionalized surface [78]. Mass-based biosensors are typically categorized into two major types which are the bulk acoustic wave resonators (BAW) or quartz crystal microbalance (QCM) and surface acoustic wave resonators (SAW) [38]. The major advantage of QCM-based biosensors is their ability to track shifts in mass in sub-nanogram amounts which can be recognized by the resonant frequency of quartz crystal. In addition, the QCM biosensor is typically used with high sensitivity for quantification of the whole cell of microorganisms [79]. The use of a QCM-based biosensor for the detection of *Escherichia coli O157:H7* was reported by Liu et al. in 2007, the detection limit was 10^2^ CFU/mL and the analysis time was less than 1.5 h [80]. Salmain et al. developed a label-free immunosensor for the rapid detection and quantification of *staphylococcal enterotoxin A* (SEA) in buffered solutions [81]. By using the quartz crystal microbalance with dissipation (QCM-D) as a transduction method, the detection limit of 20 ng/mL and analysis time was 15 min. Bayramoglu et al. developed a QCM-aptasensor for the detection of *Brucella melitensis* in milk and milk products and the detection limit was 10^3^ CFU/mL [82].

## 3. Giant Magnetoresistance (GMR)

### 3.1. GMR Effect and Devices

**Figure 3:**
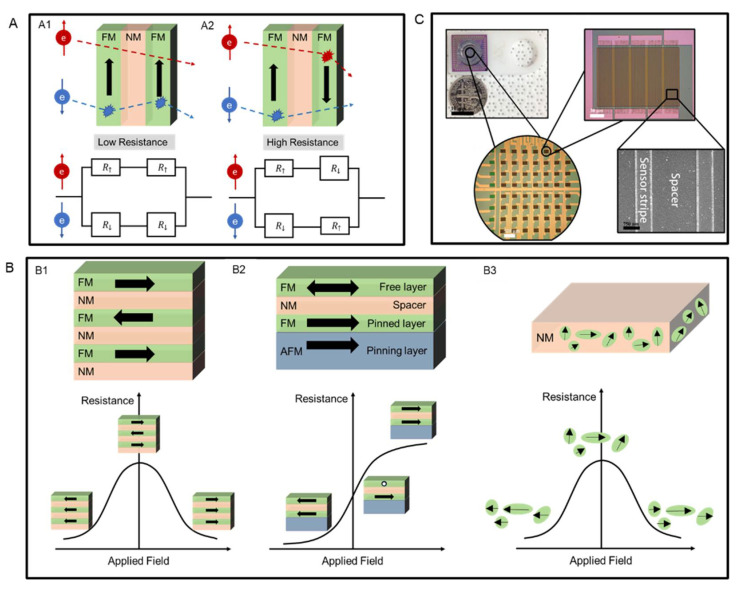
(**A**) Schematic diagram of the two-current model for spin channels in (**A1**) parallel magnetization states and (**A2**) antiparallel magnetizations states, (**B**) Schematic views of (**B1**) GMR multilayer structure, (**B2**) spin-valve structure, and (**B3**) granular structure, and their corresponding change of resistance in response to applied field curves. (**C**) A typical GMR chip consists of an 8 × 8 sensor assay. (**C**) is reprinted with permission from Ref. [89], under the terms of the Creative Commons Attribution License (CC BY).

GMR effect originates from spin-dependent scattering [83]. It is usually described by a two-channel model, as shown in Figure 3A [84]. In a layered magnetic structure, when the magnetization directions in two adjacent ferromagnetic (FM) layers are aligned (see Figure 3(A1)) to the up direction, the spin-up electrons can pass through with little scattering, while spin-down electrons are scattered strongly within both layers. This leads to a low resistance state of the whole structure. On the contrary, if magnetization directions of two neighboring FM layers are antiparallel-aligned (see Figure 3(A2)), both spin-up and spin-down electrons pass through with strong scattering in one of the two FM layers, resulting in a high resistance state.

The GMR effect was firstly found in the magnetic multilayers. It is a structure that consists of several alternating FM layers and nonmagnetic (NM) spacer layers, as shown in Figure 3(B1). Under zero applied magnetic field, the magnetization directions of every two adjacent FM layers are set to be antiparallelly aligned, which leads to a high resistance state of the system. If a large magnetic field is applied, the magnetizations in all FM layers will be aligned to the field direction, and this results in a low resistance state. The second GMR structure is the spin-valve structure consisting of two FM layers, an NM spacer layer, and an antiferromagnetic (AFM) layer, as shown in Figure 3(B2). The AFM layer (pinning layer) is used to fix the magnetization direction of the neighboring FM layer (pinned layer) through the exchange bias effect [85]. The other FM layer is called the free layer since its magnetization direction can rotate freely under an applied magnetic field. In general, to achieve better linearity, the relative magnetization directions between the pinned and free layers are set to be 90° under zero applied field.

Besides layered structures, magnetic granular structures can also exhibit the GMR effect. In 1992, Berkowitz et al. and Xiao et al. reported the GMR effect in granular Co-Cu alloys for the first time [86,87]. A granular magnetic alloy generally consists of magnetic granules and a nonmagnetic metal matrix, as shown in Figure 3(B3). Those granules have randomly distributed magnetic moments in the absence of an applied field. Therefore, the system has zero macroscopic magnetization, and the resistance is in the maximum state. When a large magnetic field is applied, the magnetic moments of those granules are aligned to the field direction, and the resistance of the system reaches its minimum state.

Among these three different GMR systems, the spin valve structure is mostly used in biosensors. Although the multilayer structure has the advantage of a high GMR ratio, it often requires a large working field. Besides, the resistance change in response to the applied field for a multilayer structure is usually nonlinear. As for the granular structure, it only has limited application due to the small magnitude of the GMR ratio in low applied fields [88]. Therefore, the spin valve structure with a low saturation field and good output linearity is the best choice for building up biosensors. Benefiting from the development of nanofabrication techniques, an individual GMR sensor is often in the size of the micrometer range, which enables hundreds to thousands of GMR sensors to be integrated on a single GMR chip. A typical GMR biosensing chip is shown in Figure 3C.

### 3.2. GMR Biosensors: Surface Modifications and Bioassay Strategies

As is discussed in the introduction section, the GMR biosensing platform uses MNPs (or magnetic beads) to label the biological analytes. In this way, the number of biomarkers can be counted by quantifying the number of MNPs, which can be realized by using a GMR sensor. Since only MNPs in the vicinity of the sensor surface can affect the MR of the sensor, properly immobilizing magnetically labeled biologic analytes to the sensor surface is essential for conducting sensitive detection [90,91,92]. This immobilization is generally realized by chemical modifications of the sensor surface followed by the establishment of biological assays.

**Figure 4:**
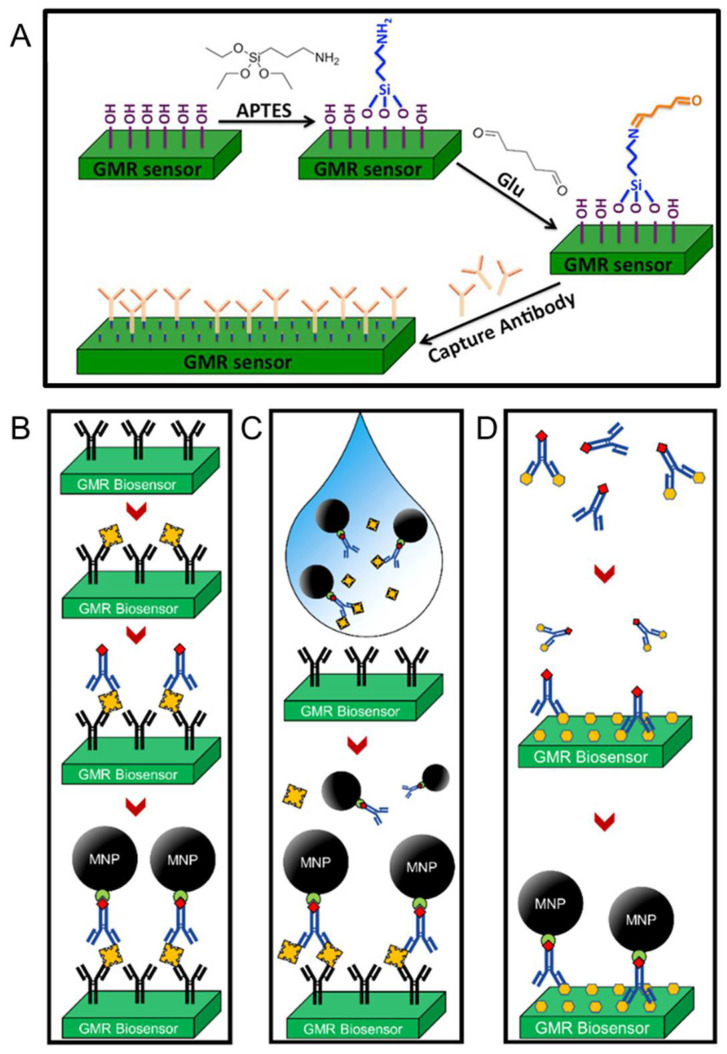
Schematic diagram of (**A**) surface modification and capture probe conjugation via the APTES/Glu method, (**B**) establishment of a traditional sandwich bioassay, (**C**) establishment of a wash-free sandwich bioassay, and (**D**) establishment of a competitive bioassay. (**A**) is reprinted with permission from Ref. [89], under the terms of the Creative Commons Attribution License (CC BY). (**B**–**D**) are reprinted with permission from Ref. [34], Copyright 2022, American Chemical Society.

In practice, one of the most used surface functionalization methods to immobilize capture probes is the 3-aminopropyltriethoxy silane (APTES)/glutaraldehyde (Glu) [APTES/Glu] method [93]. A schematic illustration of this functionalization method is presented in Figure 4A. Before the modification, the sensor surface was often natively functionalized with hydroxyl groups during the cleaning steps or pre-treatment steps. Thus, soaking the sensor in APTES solutions allows the silanol groups in APTES to connect to the surface by forming the Si-O-Si bonds. After adding the Glu solution, the amino groups exposed on the salinized surface will covalently bind to one of the two terminal aldehyde groups in Glu. The other aldehyde group on the sensor surface will then allow the binding of amino groups in capture probes.

Once the capture probes have been immobilized onto the sensor surface, different bioassays can be built up on top to conjugate the magnetically labeled biomarkers. For example, Figure 4C shows the protocol and the structure of a traditional sandwich bioassay. The target analytes are often antigens and can be bonded between capture antibodies and detection antibodies via specific antibody-antigen reactions. Then the streptavidin-coated MNPs are bound to detection antibodies through streptavidin-biotin conjugations. Although the robustness of this bioassay strategy has been demonstrated by several previous studies, it is quite time-consuming. Redundant washing steps are required after each incubation process to remove unbonded antibodies and antigens. To simplify the bioassay preparation procedure, a wash-free bioassay has been proposed, as shown in Figure 4D. During the establishment of a wash-free bioassay, detection antibodies, target analytes, and MNPs are mixed first before adding the mixture to the sensor surface. While this one-step strategy greatly improved the efficiency of building up a bioassay, the sensitivity was lower than the traditional sandwich strategy due to the increased possibility of non-specific binding [94].

Figure 4 shows another bioassay approach that is based on the competition of labeled and unlabeled detection probes and is therefore called competitive assay [32]. This method is good for detecting small analytes with limited binding sites. To conduct this approach, excessive detection probes are mixed with an analytes-containing biological sample, which leaves a mixture consisting of both occupied and unoccupied detection probes. The mixture is then added to the sensor surface that is covered with analytes. With the previous bound detection probes being washed away, unbound ones will be left on the sensor surface. In this way, the concertation of target analytes in the sample is inversely proportional to the strength of the sensor signal. Besides the aforementioned bioassay strategies, other methods such as the direct bioassay and DNA-based bioassay have also been demonstrated and reported, which further enhances the generalizability of GMR biosensors.

### 3.3. Portable GMR Bioassay Platforms for Field Test

**Figure 5:**
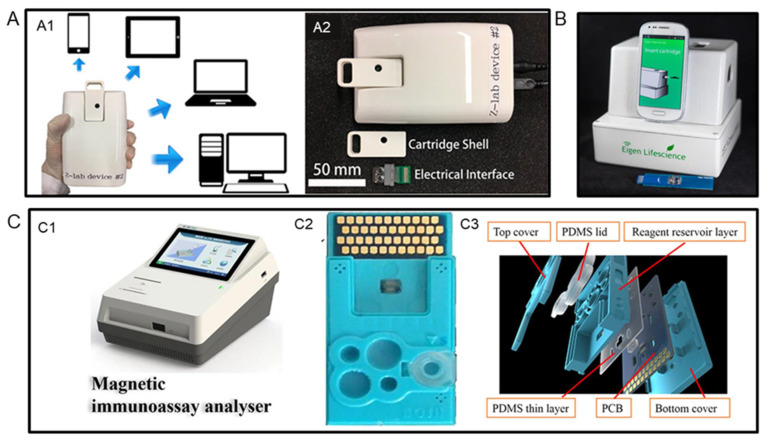
(**A**,**A1**) Z-lab can communicate with smartphones, tablets, laptops, and desktop computers. (**A2**)Photograph of the Z-lab diagnosis platform.(**B**) Photograph of the Eigen Diagnosis Platform. (**C**,**C1**) Photographs of the point-of-care GMR biosensing platform reported by Gao et al. (**C2**) Photograph of the test card. (**C3**) The structure of the test card. (**A**) is reprinted with permission from Ref. [96], Copyright 2017, American Chemical Society. (**B**) is reprinted with permission from Ref. [95], Copyright 2016, Elsevier B.V. (**C**) is reprinted with permission from Ref. [23], Copyright 2018, Elsevier B.V.

By virtue of the miniaturization of GMR sensors, they have great potential in developing point-of-care (POC) devices for the field test. To date, several research groups have reported the portable GMR biosensing platforms. For example, Choi et al. reported a potable GMR biosensing platform named Eigen Diagnosis Platform (EDP) in 2016 [95]. As shown in Figure 5B, the EDP consists of a reader station, a smartphone interface, and a disposable cartridge. By building up a wash-free multiplexed bioassay, EDP was demonstrated to have sensitivities of 0.07 and 0.33 nM for human immunoglobulin G and M antibodies, respectively. The cost per test was calculated to be less than USD 4.

In 2017, Wu et al. reported a GMR handheld system called Z-Lab [96]. As presented in Figure 5(A2), this Z-Lab platform consists of a handheld reading device, a plastic cartridge, and an electrical interface. This device can wirelessly communicate with apps installed on smartphones, tablets, and laptops, or connect to desktops via a USB connection (see Figure 5(A1)). The performance of the Z-Lab platform was evaluated by performing the detection of the influenza A virus (IAV). The LOD was found to be 15 ng/mL and 125 TCID_50_/mL for IAV nucleoprotein and 50 μL purified influenza A virus strain A variant (H3N2v), respectively. Meanwhile, the total readout time was less than 10 min and real-time data collection was allowed.

Another example of portable GMR biosensing platforms was presented by Gao et al. in 2019 [23]. Compared to the previous two examples, this platform took advantage of microfluidic technologies to enable automatic sample handling. An analyzer was built to read out the signals, and a microfluidic test card was created to process the biological sample. Their structures are shown in Figure 5C. The GMR chip matched with this platform has 40 individual GMR sensors on it. By establishing different biosensors, the portable sensing platform was proven to be able to detect 12 different tumor biomarkers simultaneously. Using the same sensing platform, Meng et al. realized the detection of N-terminal pro-B-type natriuretic with a LOD of 5 pg/mL and C-reactive protein with a LOD of 1 ng/mL, which shows the universal applicability of this platform [97,98].

## 4. GMR for Food Safety Applications

### 4.1. GMR for Foodborne Pathogen Detection

**Figure 6:**
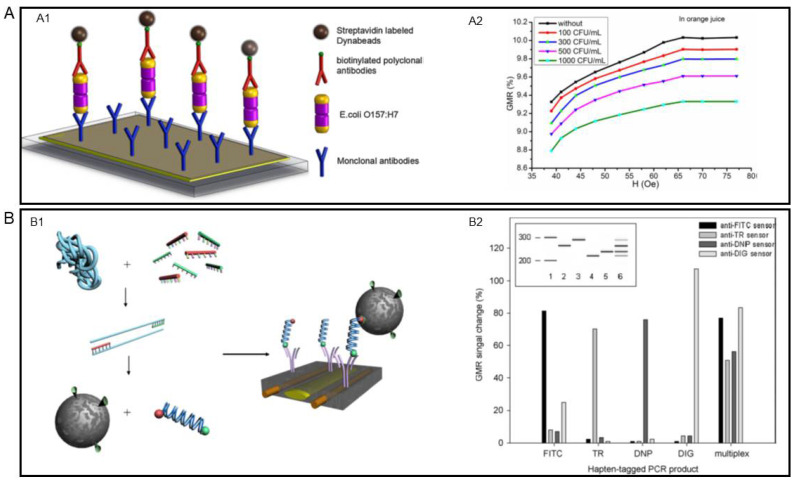
(**A**,**A1**) Schematic view of the sandwich assay for *E. coli O157H: H7*. (**A2**) The MR curves of the GMR biosensing system from detecting inactivated *E. Coli O157:H7* in orange juice. (**B**,**B1**) Detection of PCR product using the one-step assay. (**B2**) Multi-analyte detection results of Salmonella antibiotic resistance genes in a one-step assay format. (**A**) is reprinted with permission from Ref. [99], Copyright 2016, Elsevier B.V. (**B**) is reprinted with permission from Ref. [100], Copyright 2009, Elsevier B.V.

Foodborne pathogens, including bacteria, viruses, and fungi, are health-threatening and even fatal to human beings. Thus, accurate detection of these pathogens is crucial in preventing foodborne diseases. It has been proved by many research groups that GMR biosensors are capable of sensitively and rapidly detecting foodborne pathogens of different kinds. In Table 2, we summarized examples of GMR biosensors for foodborne pathogen and other food-related biomarkers detection reviewed in this section. An early-stage attempt of detecting *Escherichia coli (E. coli) O157:H7*, one of the most common foodborne bacteria, via GMR biosensor was performed by Mujika et al. in 2008 [15]. They used a multilayer GMR sensor and incorporated a microfluidic channel into the sensor to guide the biological sample. Later, Sun et al. also developed a GMR-based sensing platform that successfully detected *E. coli O157:H7* [99]. Unlike the traditional GMR sensor structure, in which the surface functionalization and bioassay conjugation happen on the sensor surface, Sun et al. built the bioassay onto an Au-coated glass slide to create a separated sample, as shown in Figure 6(A1). By covering the separated sample on the sensor surface, they achieved a LOD of 100 CFU/mL for inactivated *E. coli O157:H7* in orange juice. The detecting results are presented in Figure 6(A2).

While the targeted analytes are mostly antigens and the bioassays are established based on antibody-antigen interactions, such as the examples discussed so far, the DNA of the pathogens can also serve as analytes. For example, Koets et al. used PCR-amplified DNA as the analytes and realized a multi-analyte detection of the food pathogen *Salmonella* [100]. As shown in Figure 6(B1), the DNA genome was first amplified by PCR and then the amplicons are bound to the sensor surface. Two different assays were adept in this study. For the one-step assay, MNPs and the amplicons were mixed for 1 min, then the mixture was applied to the sensor. For the two-step assay, the DNA amplicons were firstly added to the antibody-coated sensor surface and incubated for 30 min, then unbound DNA strands were removed before adding MNPs. The LODs of the one-step and two-step assay were found to be 4% and 5% GMR signal change, respectively. The multiplexed detection results are presented in Figure 6(B2), which consists of the simultaneous detection results of four different kinds of *Salmonella* antibiotic resistance genes. The ability to conduct multiplexed detection is a key advantage of GMR biosensors compared to traditional detection methods, such as culturing and PCR. Benefiting from the development of micro- and nano-fabrication technology, multiple sensors can be integrated on a single chip. By decorating each chip with different capture probes, multiplexed detection is readily achieved.

### 4.2. GMR for Foodborne Toxin Detection

**Figure 7:**
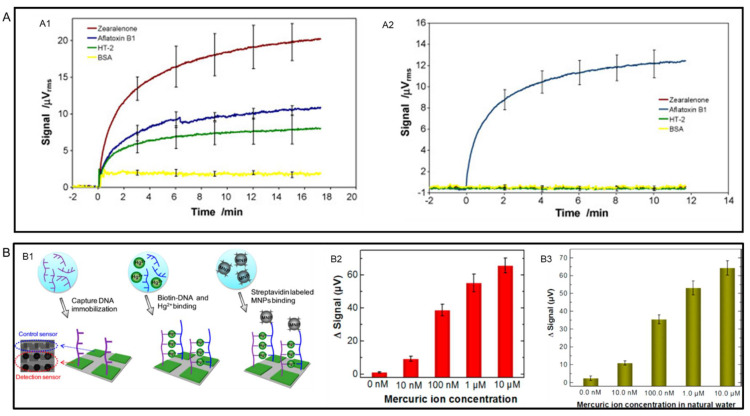
(**A**,**A1**) Real-time binding curves of AFB1, zearalenone and HT-2 at 33 ng/mL concentration. (**A2**) Cross-reactivity detection results of anti-AFB1, anti-zearalenone and anti-HT-2. (**B**,**B1**) Schematic diagram of the DNA assay for Hg^2+^ detection. (**B2**) Average signals for mercuric ions with different concentrations in a buffer. (**B3**) Average signals for mercuric ions with different concentrations in natural water. (**A**) is reprinted with permission from Ref. [101], Copyright 2010, Elsevier B.V. (**B**) is reprinted with permission from Ref. [102], Copyright 2014, American Chemical Society.

Instead of directly detecting the antigens and genomes of foodborne pathogens, an alternative way to confirm their existence is by detecting the toxins generated by those pathogens. In 2009, Mak et al. reported a multiplexed detection of mycotoxins by using a single GMR chip consisting of an 8 × 8 sensor array [101]. To demonstrate the ability of multiplexed detection, they functionalized the GMR chip with a mixture of three common mycotoxins, aflatoxins B_1_(AFB_1_), zearalenone, and HT-2. The real-time readout results of mixtures of 33 ng/mL are presented in Figure 7(A1). The specificity of GMR biosensors was also confirmed in this example. As shown in Figure 7(A2), for sensors conjugated with only anti-AFB_1_ antibodies, samples containing zearalenone, HT-2 and bovine serum albumin (BSA) gave negligible signals.

Although all the aforementioned food safety issues are related to organic contaminates, inorganic contamination can also be a severe problem, especially heavy metals contamination in water and soil [73]. The ability of GMR sensors in detecting heavy metal ion Hg^2+^ has been demonstrated by Wang et al. [102] To detect Hg^2+^ ions sensitively and selectively, they specially designed DNA strands to capture Hg^2+^ ions by forming the thymine-Hg^2+^-thymine (T-Hg^2+^-T) pair, as shown in Figure 7(B1). The detection was conducted in both buffer and natural water and the results are shown in Figure 7(B2,B3). The LOD was found to be as low as 10 nM.

### 4.3. GMR for Other Food-Related Biomarker Detection

**Figure 8:**
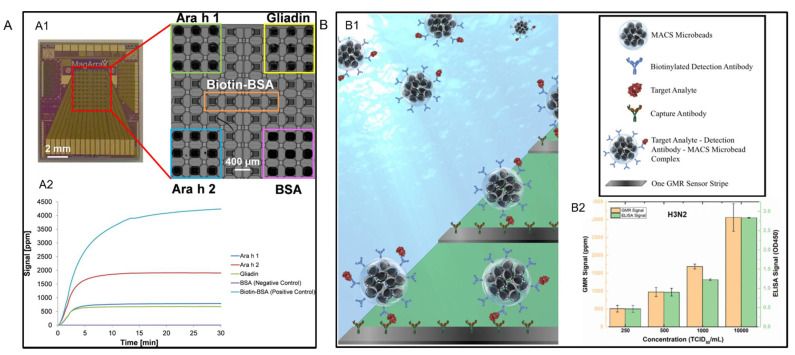
(**A**,**A1**) Image of the GMR sensor array chip with multiplexed bioassay. (**A2**) Real-time binding curve of 3 different allergens and the control groups. (**B**,**B1**) Schematic diagram of the wash-free bioassay. (**B2**) The detection of H_3_N_2_ via GMR sensor and ELISA. (**A**) is reprinted with permission from Ref. [104], Copyright 2016, Elsevier B.V. (**B**) is reprinted with permission from Ref. [103], under the terms of the Creative Commons Attribution License (CC BY).

Besides foodborne pathogens and toxins, food allergies are also health threatening. Sensitive detection of multiple food allergens was successfully conducted by Ng et al. via a GMR sensing platform [104]. They fabricated a chip with an array of 8 × 10 GMR sensors on it. As shown in Figure 8(A1), for peanut allergen Ara h 1 and Ara h 2, and wheat allergen Gliadin, each was dispensed on nine sensors. This enabled a multiplexed detection of these three different allergens on a single chip. Meanwhile, parts of the sensor array were spotted with biotin-BSA and BSA as positive and negative control groups. The real-time detection results of the GMR sensor array were shown in Figure 8(A2), which demonstrated its simultaneous detection ability of multiple allergens.

Another aspect related to food safety is preventing the spread of the virus among livestock. The detection of livestock virus has also been reported. Su et al. further developed a wash-free bioassay (see Figure 8(B1)) and successfully detected influenza A virus in swine nasal swab samples via a GMR biosensing platform [103]. They characterized the swine nasal swab samples, which were spiked with different concentrations of purified IAV H3N2v, via both their GMR biosensing platform and the ELISA. From testing results presented in Figure 8(B2), it can be concluded that the signal increased with increasing concentration for both the GMR sensing platform and ELISA. The LOD of the sensor was defined as the concentration at which the sensor signal is twice as large as the background noise level and was found to be 250 TCID_50_/mL.

## 5. Conclusions

Foodborne illness and food waste cause significant threats to human health, the environment, and the economy. Therefore, the rapid and sensitive detection of foodborne pathogens has become the top priority in food safety. Various technologies have been developed to detect foodborne pathogens. The traditional methods to detect foodborne pathogens are different based on the different requirements. The most common and widely used method is the cell culture and colony counting. However, this method is tedious, requires a lot of time, and limits the extensive application in reality. Nowadays, the development of foodborne pathogen detection technology is moving rapidly from traditional cell culture to modern detection technology, including immunological methods such as enzyme-linked immunosorbent assay (ELISA). These include Immunomagnetic separation (IMS) assay, metabolic technology such as PCR, DNA probe, microcalorimetry, biosensor-based methods such as optical biosensors, electrochemical biosensors, and piezoelectric biosensors, as well as giant magnetoresistance (GMR) biosensors, and so on [10,64,74,81,105,106,107,108]. Although sophisticated techniques such as the immunological ELISA assays and PCR-based assays provide sensitivity and conclusive results, biosensors allow a superior or comparable sensitivity at a lower cost and time, and with less operator training. Along with the development of nano- and micro-fabrication technologies, high-density GMR sensor arrays have been achieved on a single chip of a size no larger than a fingernail. The small size of the GMR chip makes it compatible with portable devices and therefore has great potential in developing point-of-need sensing platforms. Integrating multiple sensors on one chip also enables multiplexed detection of different biomarkers at the same time. This simultaneous detection ability can reduce the detection time to a large extent.

## 6. Future Prospects

Although the robustness of GMR biosensors has been demonstrated in many examples, the current GMR biosensing platform can be further optimized in several ways. To date, most of the studies on GMR biosensors are focusing on processed samples, such as purified proteins. While using processed sample is acceptable in laboratory settings, field test requires the direct direction of unprocessed samples. The current wash-free bioassay strategy can contribute to this end by decreasing assay time and simplifying operation protocols, but the sensitivity may need to be further improved. The integration of microfluidic systems has brought new possibilities for handling unprocessed samples. The microfluidic systems can be used to pump liquid samples and filter unrelated substances [109]. This could reduce the assay time to a large extent. Challenges to integrating microfluidic systems into GMR sensors lie in the binding process and manipulating MNPS behaviors in the microfluidic channel [32].

Combining microfluidic systems and wash-free bioassay strategies, a fully automatic lab-on-a-chip GMR sensing platform can be readily achieved. With such a platform, point-of-detection of various biomarkers related to food safety is realizable even for untrained personnel. Besides common pathogens and toxins, these sensors may have the potential for further applications in detecting other samples including pesticides and veterinary drug residues.

## Figures and Tables

**Table 1:** Advantages and Disadvantages of Different Foodborne Pathogen Detection Methods.

Technique	Advantages	Disadvantages
Traditional Culture and Colony-based Methods	Low cost High sensitivity High credibility	Time-consuming Labor-intensiveness
Polymerase Chain Reaction (PCR)	High sensitivity High specificity High accuracy	Nucleic acid extraction Expensive equipment Requiring trained technicians No distinction between dead or live cells
Immunological Methods	High sensitivity High specificity Real-time analysis	Requiring trained technicians Expensive
Optical Biosensors	Easy to use Miniaturizable Low costs High sensitivity	Need for high energy sources Interference of incident light Narrow concentration range
Electrochemical Biosensors	Rapid Simple operation Miniaturizable	Low selectivity
Mechanical Biosensors	High sensitivity Rapid Simple operation Stable output	Low sensitivity with liquid samples Interference induces by nonspecific binding

**Table 2:** Summary of GMR Biosensors for Food-related Biomarkers Detection.

MNPs	Target Analytes	Matrices	Assay Time	LOD	Detection Range	Ref.
Dynabeads	*E. coli O157:H7*	Pure culture	N/A	N/A	N/A	[15]
Streptavidin-coated magnetic particles	LamB gene of *E. coli*	Pure culture	3 min	4 pM	4 to 250 pM	[100]
MACS	Aflatoxins B_1_	Pure culture	15 min	50 pg/mL	N/A	[101]
MACS	Zearalenone	Pure culture	15 min	50 pg/mL	N/A	[101]
MACS	HT-2	Pure culture	15 min	50 pg/mL	50 pg/mL–50 ng/mL	[101]
Dynabeads	*E. coli O157:H7*	Orange juice	N/A	100 CFU/mL	N/A	[99]
MACS	Mercuric ion	Natural water	30 min	10 nM	10 nM to 10 μM	[102]
MACS	IAV nucleoprotein	Pure culture	N/A	0.3 nM	N/A	[103]
MACS	IAV H3N2v	IAV-spiked nasal swab	N/A	250 TCID_50_/mL	N/A	[103]
MACS	Ara h 1	Pure culture	15 min	7.0 ng/mL	7.0 to 2000 ng/mL	[104]
MACS	Ara h 2	Pure culture	15 min	0.2 ng/mL	0.2 to 250 ng/mL	[104]
MACS	Gliadin	Pure culture	15 min	1.5 ng/mL	1.5 to 4000 ng/mL	[104]

MACS stands for streptavidin-coated superparamagnetic MNPs produced by Miltenyi Biotec Inc., San Jose, CA, USA.
